# Characterization of the complete chloroplast genome of *Euphorbia helioscopia* Linn. (Euphorbiaceae), a traditional Chinese medicine

**DOI:** 10.1080/23802359.2019.1682480

**Published:** 2019-10-24

**Authors:** Peng Li, Xiaoqing Liang, Xiaoni Zhang

**Affiliations:** Department of Pharmacy, Xi’an International University, Xi’an, Shaanxi, China

**Keywords:** *Euphorbia helioscopia*, Euphorbiaceae, chloroplast genome, Illumina sequencing, phylogenetic analysis

## Abstract

*Euphorbia helioscopia* Linn. known as a traditional Chinese medicine of Euphorbiaceae, which contains terpenes, steroids, flavonoids, acetophenones, tannins, phenylpropanoids, cerebrosides and so on. *Euphorbia helioscopia* L. was used to treat malignant tumors and chronic obstructive pulmonary diseases such as cough, phlegm-turbidity, asthma, and chronic bronchitis. The complete chloroplast genome was assembled by Illumina paired-end reads data. The length of circular cp genome distribution in 160,041 bp, containing a large single-copy region (LSC) of 88,832 bp, a small single-copy region (SSC) of 17,145 bp and a pair of inverted repeat (IR) regions of 27,032 bp. In addition, 11 genes possess a single intron, while the other two genes (*ycf3*, *clpP*) have a couple of introns. The GC content of entire *Euphorbia helioscopia* L. cp genome, LSC, SSC and IR regions are 35.9, 33.1, 30.3, and 42.3%, respectively. From the NJ phylogenetic tree analysis showed that *Euphorbia helioscopia* L. and *Euphorbia esula* are closely related to each other within the family Euphorbiaceae.

*Euphorbia helioscopia* Linn. is a traditional Chinese medicine of Euphorbiaceae, which contains terpenes (iridoids, sesquiterpenoids, diterpenoids, triterpenoids), steroids, flavonoids, acetophenones, tannins, phenylpropanoids, cerebrosides and so on (Shi et al. 2008; Wang et al. 2018). *Euphorbia helioscopia* L. was used to treat malignant tumors and chronic obstructive pulmonary diseases such as cough, phlegm-turbidity, asthma and chronic bronchitis (Chen et al. 1979; Cheng et al. 2015). Modern pharmacological research shows that Euphorbia diterpenoids have anti-cancer effects (Wang et al. 2018).

The complete chloroplast (cp) genome is a conserved structure of four parts, the four parts as follows, a pair of inverted repeats (IRs), separated by a large single-copy region (LSC) and a small single-copy region (SSC) (Wolfe et al. 1992; Lee et al. 2007). Our study will be very important for futher studying the phylogenetic relationships between *Euphorbia helioscopia* L. and Euphorbiaceae.

The fresh leaves of *Euphorbia helioscopia* L. were collected in the Botanical Garden of Northwest University (34°16′N, 108°54′E; Shaanxi, NW China). A voucher specimen (FS190321) was deposited at the structural plant laboratory in International University. We used the modified CTAB method to extract the genomic DNA (Doyle and Doyle 1987) and then followed the manufacturer’s specification of Illumina HiSeq X Ten Sequencing System (Illumina, San Diego, CA) to construct a shotgun library.

The *Euphorbia esula* (GenBank: KY000001) was used as the initial reference to assemble cp genome with the program MITObim v 1.8 (https://github.com/chrishah/MITObim) (Hahn et al. 2013). Based on the web-based tool OGDRaw v1.2 (http://ogdraw.mpimp-golm.mpg.de/) to generate the complete cp genome (Lohse et al. 2013). The complete cp genome sequence has been submitted to GenBank (accession number MNI 199031).

The complete cp genome is a circular double-stranded DNA molecule with a typical quadripartite structure, including a pair of IRs, an LSC region, and an SSC region. Based on the sequencing results, we got 33,132,728 raw Paired-End Reads, the length distribution in 160,041 bp (GC content accounts for 35.9%). In addition, the length of LSC region, SSC region and IRs regions were distributed as 88,832 bp (GC, 33.1%), 17,145 bp (GC, 30.3%), and 27,032 bp (GC, 42.3%), respectively. It encodes 113 complete genes, containing 77 protein-coding genes, 32 transfer RNA genes, and 4 ribosomal RNA genes. 5 tRNA genes (*tRNA-Ala, -Ile, -Leu, -Lys,* and *-Val*) harbor a single intron. *AtpF*, *ndhA*, *ndhB*, *rps12*, *rpl2,* and *rpoC1*, these 6 PCG genes possess a single intron, *clpP* and *ycf3* harbor two introns, while, 69 PCG genes no intron.

To study the phylogenetic position of *Euphorbia helioscopia* L. we constructed a neighbour-joining (NJ) phylogenetic tree (Figure 1) through MEGA7 with 1000 bootstrap replicates (http://www.megasoftware.net/) based on the concatenated coding sequences of 13 chloroplast PCGs for 12 plastid genomes from published species of Euphorbiaceae (Kumar et al. 2016). From Figure 1, we find that *Euphorbia helioscopia* L. (MNI199031) is closely related to *Euphorbia esula* (GenBank: KY000001).

**Figure 1.**
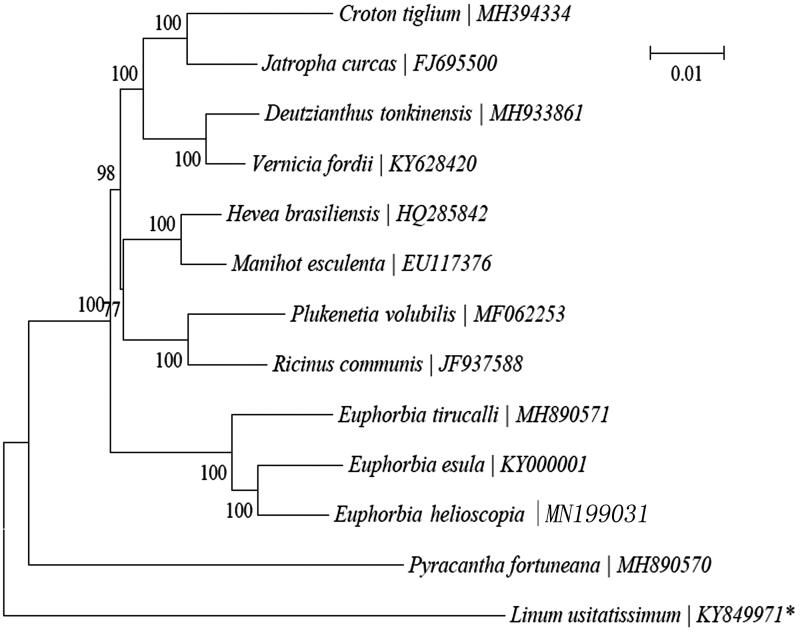
Phylogeny of 13 species within the order Euphorbia based on the neighbour-joining (NJ) analysis of chloroplast PCGs. The bootstrap values were based on 1000 resamplings and are placed next to the branches. *represents the outgroup of Euphorbia.
